# Early special educational needs provision and its impact on unplanned hospital utilisation and school absences in children with isolated cleft lip and/or palate: a demonstration target trial emulation study protocol using ECHILD

**DOI:** 10.3310/nihropenres.13472.1

**Published:** 2023-10-24

**Authors:** Vincent G Nguyen, Kate M Lewis, Ruth Gilbert, Lorraine Dearden, Bianca De Stavola

**Affiliations:** 1 Institute of Child Health, University College London, London, England, WC1N 1EH, UK; 2 Social Research Institute, University College London, London, England, WC1H 0AL, UK

**Keywords:** Special Educational Needs, Trial Emulation, Confounding-by-indication, Cleft Lip and/or Palate

## Abstract

**Background:**

Special educational needs (SEN) provision is designed to help pupils with additional educational, behavioural or health needs; for example, pupils with cleft lip and/or palate may be offered SEN provision to improve their speech and language skills. Our aim is to contribute to the literature and assess the impact of SEN provision on health and educational outcomes for a well-defined population.

**Methods:**

We will use the ECHILD database, which links educational and health records across England. Our target population consists of children identified within ECHILD to have a specific congenital anomaly: isolated cleft lip and/or palate. We will apply a trial emulation framework to reduce biases in design and analysis of observational data to investigate the causal impact of SEN provision (including none) by the start of compulsory education (Year One – age five year on entry) on the number of unplanned hospital utilisation and school absences by the end of primary education (Year Six – age ten/eleven). We will use propensity score-based estimators (inverse probability weighting (IPW) and IPW regression adjustment IPW) to compare categories of SEN provision in terms of these outcomes and to triangulate results obtained using complementary estimation methods (Naïve estimator, multivariable regression, parametric g-formula, and if possible, instrumental variables), targeting a variety of causal contrasts (average treatment effect/in the treated/in the not treated) of SEN provision.

**Conclusions:**

This study will evaluate the impact of reasonable adjustments at the start of compulsory education on health and educational outcomes in the isolated cleft lip and palate population by triangulating complementary methods under a target-trial framework.

## Plain English summary

Children born with cleft lip and/or palate have been shown to have lower academic performance compared to the general population and have also been shown to have higher attendances to hospitals. To support children with such health and education needs, special educational needs provisions such as teaching assistants can be provided. The aim of this study is to understand whether children with cleft lip and/or palate were better off on average in receiving special education needs at the start of primary school in terms of hospital usage and school absences.

## Introduction

Special educational need (SEN) provision offers reasonable adjustments in children and young people (CYP) in an educational environment who need additional health, academic, or behavioural support. This includes children with complex health requirements or learning difficulties. SEN provision offers support to those with needs using a variety of facilities. SEN provision in the English educational setting is divided into two main categories: SEN Support (previously known as Action, Action Plus or non-Statemented SEN) and Education and Health Care Plan (EHCP, this is previously known as a statement of SEN) ( Timpson & Department for Education, 2014). SEN Support is organised by the educational environment (e.g., school or college) and provides access to children and young people in need of SEN provision, with support that may include teaching assistants who aid in communications, specialised adapted learning programmes and supporting physical needs. An EHCP is funded by local authorities for children and young people who require further adjustments and often require additional health specific resources (compared to SEN Support) to aid in education, health, and social care needs. Due to the funding and organisational streams of SEN provision, allocation of SEN provision varies over time and location, which can be impacted by changes in legislation, school governance structure and local authority ( Liu *et al.*, 2020).

Currently, there is limited research on the impact of SEN provision on academic and healthcare outcomes in populations who have a need for SEN provision. To estimate the causal effect of SEN provision on outcomes, randomised controlled trials (RCT) would have to be conducted, however such study designs are not always feasible due to the human, time, financial and ethical costs associated. As SEN provision is universally available in primary education environments (schools teaching pupils aged five to eleven years) in England, conducting an RCT would be unfeasible and possibly unethical for certain groups of children. In lieu of RCTs, observational studies provide a pragmatic, data-driven, observational alternative when trials are not possible. One major challenge with using observational data when compared to data collected from RCTs is the risk of confounding, particularly, confounding by indication whereby assignment to treatment is not random and is often related to the severity of a medical condition ( Salas *et al.*, 1999). However, attentive study design can mitigate such biases in observational data by emulating the protocol of an equivalent RCT ( Hernán & Robins, 2016).

An observational dataset that can be used to estimate the causal effect of recorded SEN provision on healthcare outcomes is the ECHILD dataset ( Mc Grath-Lone *et al.*, 2022). The ECHILD dataset is the first dataset in England of linked academic data (National Pupil Database - NPD) with secondary care hospital data (Hospital Episode Statistics - HES) for all pupils educated in state funded schools, and has been used to investigate the associations between health, education and social care ( Mc Grath-Lone *et al.*, 2022). Therefore, the ECHILD dataset provides the opportunity to conduct observational studies with long-term follow-up with current follow-up being up to age 25 years (from birth in 1995 until hospitalisations in 2020). With data on clinical diagnoses, social care contacts, hospital visits, academic attainment, school absences and SEN provisions in school, the ECHILD dataset enables adjustment for different confounders in populations at risk and to focus on specific populations at risk, identifiable from the linked hospital data, for example using phenotypes such as children with major congenital anomalies (e.g., Down Syndrome), cerebral palsy, developmental disorder of scholastic skills, epilepsy, diabetes, and premature birth. Education data in ECHILD include provision of support for SEN, free school meal status and measures of socioeconomic deprivation, as well as national examination results (at multiple key stages) and absence and exclusions from school, while health related outcomes such as (specific types of) hospitalisations are also available via linkage to hospital records.

In this study protocol, we describe how we aim to use the ECHILD dataset to design a study that appropriately emulates an RCT to address causal question of the impact of receiving alternative categories of SEN provision (including none) on unplanned hospital utilisation and school absences in children who were born with cleft lip and/or palate (CLP). Incidences of CLP are identified in 900 new-borns in England yearly and impact communications (hearing and speech), dental health ( Gallagher & Collett, 2019) and psychosocial health. CLP is associated with reduced academic attainment ( Fitzsimons *et al.*, 2018) and has been linked to a three-fold increase in hospitalisations when compared to those without CLP for all ages ( Bell *et al.*, 2016). Previously observational evidence has suggested that extra support at the beginning of compulsory education may benefit academic outcomes of children with CLP ( Fitzsimons *et al.*, 2018). Whilst previous literature hypothesised the impact of SEN provision on academic outcomes ( Fitzsimons *et al.*, 2018), there is limited to no literature assessing the impact of SEN provision on unplanned hospital utilisation and school absences.

## Methods

### Ethics and dissemination

Research ethics committees have approved the use of the ECHILD database; access to the ECHILD database is approved by the ECHILD team, who are contactable at ich.echild@ucl.ac.uk for proposals for projects using ECHILD.Stake holder groups consisting of focus groups of young people, parents and service providers will help us frame the research question, interpret, and communicate our findings to policy makers, health and education services and families to promote translation of our findings into practice.

### Stakeholder involvement

Prior to developing this protocol, two independent meetings were conducted with stakeholders (parents, pupils, teachers) to understand which medical conditions are of interest and which entry timepoints are important for child development. The first meeting was with the Department for Education’s national young SEN advisory group (FLARE) on the 18 of September 2021 and the second with the Young Persons Advisory Group for research at Great Ormond Street Hospital on the 27 of November 2021. This engagement identified that school entry is an important key milestone when SEN provisions are required. Therefore, in the proposed study, we have used school start as our entry point and will generate further target trials based upon further patient engagement.

### Study design and setting

The study will be an observational study based on data from the ECHILD dataset previously described in ( Mc Grath-Lone *et al.*, 2022) which includes individuals born between 1 September 1995 and 31 August 2020 in England. To reduce confounding-by-indication and other forms of biases when using observational data, we will use a target trial framework to guide eligibility, entry, and an appropriate follow-up period ( Hernán *et al.,* 2022). Analyses will be conducted in the Office for National Statistics Secure Research Service using Stata ver. 17 (proprietary, StataCorp) and R ver. 4.0.2 (open source, R Foundation) and the code for the study will be made available via GitHub.

### Dataset and linkage

The data source we propose is the ECHILD database, a pseudo-anonymised dataset that links the National Pupil Database (NPD) with Hospital Episode Statistics (HES). In brief, the ECHILD's extract of the NPD contains data from academic terms (Summer, Autumn, and Spring) between 2006 and 2020 and contains information on (but not limited to) school, local authority, year/month of birth, gender, ethnicity, first language, socioeconomic status, free school meal status, absence related data, social care/children in need related data and SEN provision.

The ECHILD’s extract of HES contains details on accident and emergency attendance, admitted patient care, critical care, and outpatient appointments between 1997 until 2021. It contains details on birth admissions, sex recorded by physician, ethnicity, clinical information recorded during hospital admissions, including details of diagnoses, and operations. HES covers 99% of public hospital activity in England ( Herbert *et al.*, 2017). HES records since 1998 are also linked to ONS Mortality data covering information on causes and timing of deaths. The linkage coverage periods are described in Mc Grath-Lone *et al.* (2022). ECHILD has been shown to have a linkage rate between NPD and HES of 95%; the high linkage rates are attributable through a two-stage linkage process ( Libuy *et al.*, 2021).

Full methodology of creating the ECHILD dataset is described by Mc Grath-Lone *et al.,* 2022. Enquiries to access to the ECHILD dataset is obtainable by contacting ich.echild@ucl.ac.uk; all researchers accessing the ECHILD dataset will need to be an Office of National Statistics (ONS) accredited researcher. Access to the ECHID dataset will be made through the ONS Secure Research Service, a trusted research environment which requires the researcher’s institution to have Assured Organizational Connectivity, Population

Our population consists of children with isolated CLP recorded in hospital records and followed from Year One of school (the first full year of compulsory education, where pupils are five years old on the first day) between academic years 2008/2009 and 2018/2019 (i.e., born between 1 September 2003 and 31 August 2013). This period was chosen as it contains information of school readiness tests (known as Early Years Foundation Profile - a known good predictor of SEN provision) and 2018/19 was the last entry-into-school academic year prior to the COVID-19 pandemic where access to hospitals and provision of education vastly changed. To identify pupils who started Year One between 2008/2009 and 2018/2019, the earliest recording of “1” from the “NCActualYear” (National Curriculum Actual Year) variable in the NPD dataset will be used; for children whose “NCActualYear” variable is marked as empty or X, we will use “AgePartAtStartOfAcademicYear” equal to 5.

To identify pupils with isolated CLP, International Classification of Diseases version 10 (ICD-10) codes will be applied to primary and secondary HES diagnoses in any hospital admission prior to the start of compulsory education using the following codes: Q35*, Q36* and Q37*. For each pupil, the earliest recorded date in HES will be considered the “diagnosis” date. Pupils whose first recording of CLP in HES is after their first year in school will not be included as SEN allocation needs to proceed diagnosis to avoid reverse causality. Children born with further major congenital anomalies, will be identified using the EUROCAT code list ( ‘EUROCAT Guide 1.5 Chapter 3.3’, 2023) and excluded using the ICD-10 codes listed in Table 1 to reduce competing needs for SEN provision. The EUROCAT code list was used as it captures major congenital anomalies and not minor anomalies.

**Table 1.** Inclusion and exclusion criteria used to define isolated cleft lip and/or palate.

Condition, by severity	ICD-10 codes *	
	Inclusion	Exclusion **
Cleft palate	Q35x	Q-chapter, D215, D821, D1810 **, P350, P351, P371 Apart from: Q0461 **, Q0782 **, Q101, Q102, Q103, Q105, Q135, Q170, Q171, Q172, Q173, Q179, Q174, Q180, Q181, Q182, Q184, Q185, Q186, Q187, Q1880 **, Q189, Q2111, Q250 if gestational age <37 weeks, Q2541, Q256 if gestational age <37 weeks, Q261, Q270, Q314, Q320, Q331, Q381, Q382, Q3850 **, Q400, Q4021 **, Q430, Q4320 **, Q4381 **, Q4382 **, Q523, Q525, Q527, Q53, Q5520 **, Q5521 **, Q610, Q627, Q633, Q653-Q656, Q662-Q669, Q670-Q678, Q680, Q6821 **, Q683-Q685, Q6810 **, Q7400 **, Q752, Q753, Q760, Q7643, Q765, Q7660 **, Q7662 **, Q7671 **, Q825, Q8280 **, Q833, Q845, Q899
Cleft lip	Q36x	
Unilateral cleft lip and palate	Q371, Q373, Q375, Q379	
Bilateral cleft lip and palate	Q370, Q372, Q374, Q378	

*Identified as a primary or secondary diagnoses in any hospital admission record prior to the start of Year 1 of school (age 5 at entry); **all cleft lip and/or palate groups also have congenital anomalies (excluding those relating to cleft lip and/or palate) excluded; ICD-10 = International Classification of Diseases version 10

We will also restrict our analyses to those born in an NHS funded English hospital due to the importance of birth characteristics such as maternal age, birth weight and gestational age. Additionally, as congenital anomalies are disproportionately recorded in those born in hospital, we have further reasons to restrict our population to those with a birth record in HES to avoid misclassification of congenital anomalies.

### Intervention

The intervention will be defined by the categories of recorded SEN provision (including none) in Year one of school (ages five or six). Whilst SEN provision can change throughout a CYP’s educational journey, our implementation of trial emulation focusses on an intention-to-treat analysis (ITT) with the intervention defined at the start of compulsory education. Therefore, we are analysing the initial assignment of treatment and not whether treatment was adhered to or provided. We choose the start of compulsory education as we believe this is a population in need of SEN provision at the start of their educational journey based upon prior evidence of educational ( Fitzsimons *et al.*, 2018) and healthcare needs.

To capture differences in type of SEN provision due to severity of CLP, along with other confounders (see covariates section), we aim to classify our exposure variable as “categories of SEN provision” (None, SEN, EHCP) as opposed to a binary outcome (i.e., SEN vs no SEN). To establish SEN status at Year One, we will use the January (Spring) census in Year One of school due to funding being calculated using these censuses. See Table 2 for a list of variables describing SEN in the NPD. See Statistical Analysis section for more detail on analysing comparison groups.

**Table 2.** List of variables recording special educational needs in the National Pupil Database.

Variable Name in NPD	Variable Description as per the NPD data dictionary
PrimarySENtype	Nature of pupil's primary special educational need. For pupils with a SEN status of E or K their main or primary need and, if appropriate, their secondary need, should be recorded.
SecondarySENtype	Nature of pupil's secondary special educational need. For pupils with a SEN status of E or K their main or primary need and, if appropriate, their secondary need, should be recorded.
SENProvision	Provision types under the SEN Code of Practice
CensusSEN	Provision types under the SEN Code of Practice.
SEN_provision	Special Educational Needs provision
SENA SENELK SENELSE SENF SENPS SEN_ALL SENAPK SENSE	• SENA: Does a pupil have SEN - school action? • SENELK: Does pupil have SEN support? • SENELSE: Does pupil have SEN with statement or EHC plan? • SENF: Pupil SEN status • SENPS: Does pupil have SEN - Action Plus or Statemented? • SEN_ALL: Does pupil have SEN with or without statement or EHC plan? • SENAPK: Does pupil have SEN without statement or EHC plan? • SENSE: Does pupil have SEN with statement or EHC plan?
LatestSEN	Provision types under the SEN Code of Practice.
SENProvisionMajor	Pupil's major SEN provision group based on SEN provision code.
SENstatus	Provision types under the SEN Code of Practice.
SENUnitIndicator	Indicates if a pupil with SEN in a mainstream school is a member of a SEN Unit (sometimes called special class)
SpecialProvisionIndicator	Indicates if a pupil with SEN in a mainstream school is a member of an SEN Unit, special class or resourced provision.

NPD = national pupil database, SEN = special educational needs

**Figure 1.**
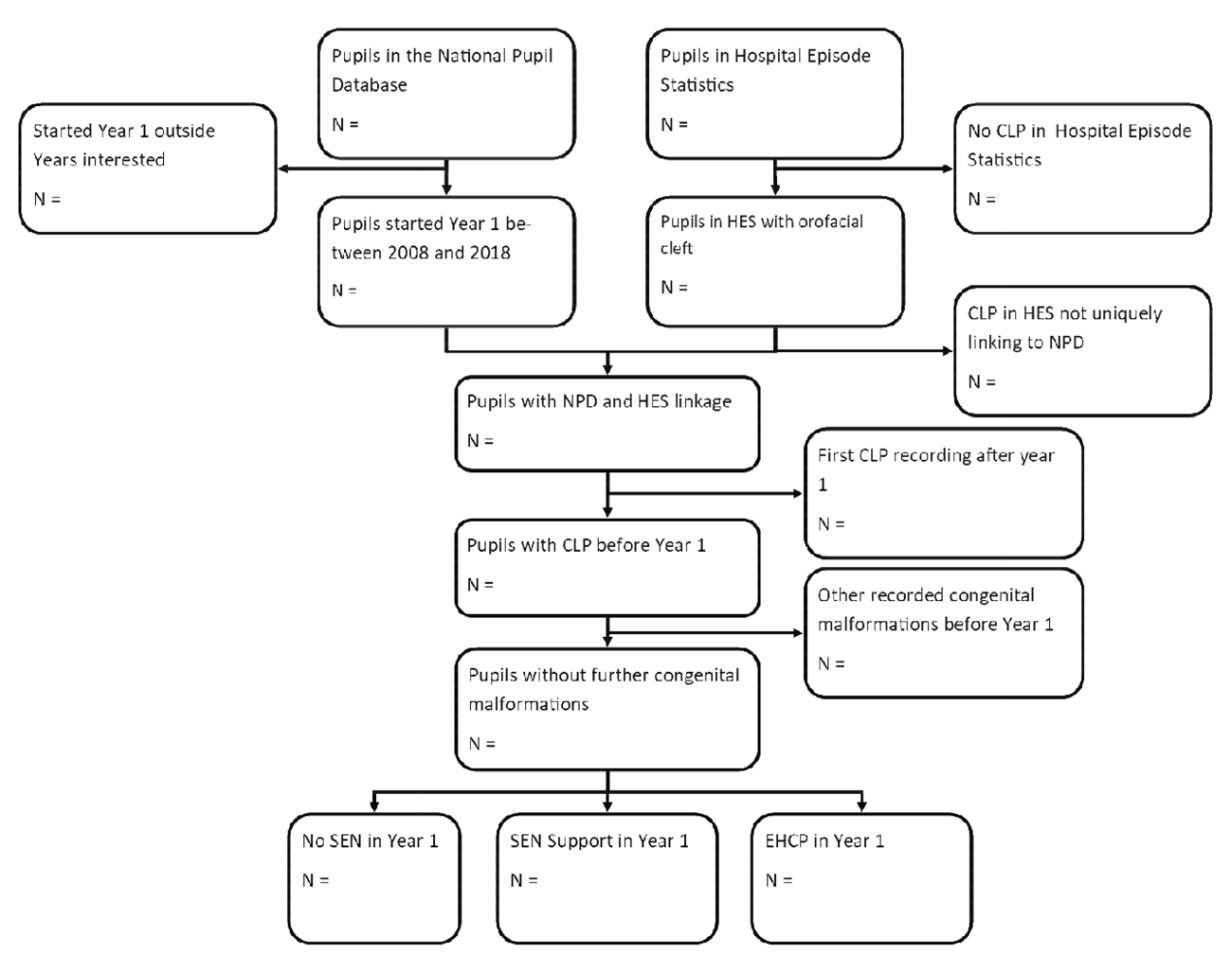
Flowchart of how the proposed population would be derived. Our population would consist of those who are identified in HES with cleft lip and/or palate before Year 1, who have a birth record in HES and who are linkable to NPD. SEN levels will be based upon SEN recorded by Year 1. No SEN represents either not having recorded SEN provision or receiving SEN after Year 1. **Children with cleft lip and/or palate (including bilateral and unilateral):** Q35x Q36x Q371, Q373, Q375, Q379, Q370, Q372, Q374, Q378. Exclusion criteria is MCA.

Figure 1 shows our planned Consolidated Standards of Reporting Trials (CONSORT) flow diagram for identifying the population and classifying it according to the exposure variables (categories of recorded SEN provision).

### Follow-up

The study population will be followed-up from the initial January census in Year One (to account for time to apply for SEN) until the end of primary school (end of July of Year Six), lost to follow-up, or end of study, whichever occurred first. Children will be considered lost to follow-up if they no longer appear in any NPD school census during primary education; this could be due to a variety of reasons including, transfer to a non-state-funded school, emigration, death, or off-rolling ( Jay *et al.*, 2022).

Although ECHILD can be used to follow up children beyond the end of primary school for some academic cohorts, we will limit our follow-up period in this protocol to Year 6 for two reasons: firstly, entering secondary (or middle) education will re-evaluate the need for SEN, and many pupils may no longer be offered SEN provision, and secondly, the time between the assignment of SEN considered here and the outcome may be too long if beyond Year Six, with the outcome affected by many intermediate factors (pupils starting Year One in 2008, would have 13 years of follow-up in HES).

### Outcome variables

The outcomes of interest include both unplanned hospital usage and school absences due to the health needs for SEN provision and the intervention being delivered in an educational setting.

Unplanned hospital usage will be measured in days between January of Year One (recorded allocation of SEN provision) and end of follow-up. To identify unplanned hospital usage in HES Admitted Patient Care, we will use the “admission method” variable of the first episode of each admission in HES (admimeth) ( Table 3 for the case definition). For hospital utilisation that did not require an overnight admission, we will use the HES Accident and Emergency dataset to account for non-admitted unplanned hospital utilisation ( Harron *et al.*, 2018). We aim to combine the “Admitted Patient Care” and “Accident and Emergency” datasets to create a timeline of unplanned hospitalisation between the January census in Year One and the end of Year Six. When an unplanned admission and recording in accident and emergency occurs on the same day, we will only count this as one day, for example when the pupil is initially presented in accident and emergency and is then admitted on the same day.

**Table 3.** Determining unplanned admissions in hospital episode statistics admitted patient care.

Value of *admimeth* variable in HES	Meaning	Action
**11, 12, 13**	Planned admission	Planned
**21, 22, 23, 24, 25,** **28, 2A, 2B, 2D**	Unplanned admission	Unplanned
**2C**	Unplanned admission for a baby born at home as intended (available from 2013/14)	Other (birth)
**31, 32**	Maternity admission	Other (maternity)
**82, 83**	Birth of a baby	Other (birth)
**81**	Transfer of any admitted patient from other Hospital Provider other than in an Unplanned	Planned
**84**	Admission by Admissions Panel of a High-Security Psychiatric Hospital, patient not entered on the HSPH Admissions Waiting List (available between 1999 and 2006)	Planned
**89**	HSPH Admissions Waiting List of a High-Security Psychiatric Hospital (available between 1999 and 2006)	Planned
**98**	Not applicable (available from 1996/97)	Other
**99**	Not known: a validation error	Other

Absences in the NPD are recorded termly as sessions, corresponding to half-days in school; the total number of potential sessions in a term is also provided. School absences will be measured as sessions between January of Year One and the end of follow up (maximum: end of Year Six). As the population of interest is based upon health needs, our school absences will include those related to medical need. See Table 4 for the case definition of medically related absences during school. In addition to medically-related absences, we will separately evaluate the impact of SEN on unauthorised absences; such evaluations are needed as absenteeism is related to poor academic performance ( Allen *et al.,* 2018).

**Table 4.** Determining medical related absences in the National Pupil database.

Absence Reason in NPD	Meaning	Action
C	Number of authorised sessions missed during the academic year as pupil is absent due to other authorised circumstances.	Exclude
E	Number of authorised sessions missed during the academic year as pupil is excluded, with no alternative provision made.	Exclude
F	Number of authorised sessions missed during the academic year due to agreed extended family holiday.	Exclude
G	Number of unauthorised sessions missed during the academic year as pupil is on a family holiday, not agreed, or is taking days in excess of an agreed family holiday.	Include
H	Number of authorised sessions missed during the academic year due to agreed family holiday.	Exclude
I	Number of authorised sessions missed during the academic year due to Illness (NOT medical or dental etc. appointments).	Include
M	Number of authorised sessions missed during the academic year due to medical/ dental appointments.	Include
N	Number of unauthorised sessions missed during the academic year as pupil missed sessions for a reason that has not yet been provided.	Include
O	Number of sessions missed during the academic year for an unauthorised absence not covered by any other code/description.	Include
R	Number of authorised sessions missed during the academic year due to religious observance.	Exclude
S	Number of authorised sessions missed during the academic year due to study leave.	Exclude
T	Number of authorised sessions missed during the academic year due to traveller absence.	Exclude
U	Number of unauthorised sessions missed during the academic year as pupil arrived after registers closed.	Exclude

We are not planning to study academic performance by the end of primary education as an additional outcome. This is motivated by the limited number of children in our population who would have recorded Key Stage Two academic outcomes (only those born between 2003–2008). With an estimated prevalence rate of 900 CLP births per year ( CRANE, 2021), of whom an estimated 62% are expected to have isolated CLP ( Fitzsimons *et al.*, 2023), we would have very small groups of children receiving (some of) the interventions. We will re-evaluate the suitability of evaluating the impact of SEN provision on academic outcomes and include this in our analyses according to the numbers with Key Stage Two data.

### Covariates

To account for non-random SEN provision assignment, we will use information on several covariates that are known or suspected to be associated with SEN provision and hospital contacts based upon prior literature. These include CLP specific influences, further clinical/birth, socio-demographic, geographical, and educational influences. See Table 5 for a list of potential confounders from relevant literature. The distribution of these potential confounders by exposure status will be examined (see Table 5 for an outline) and directed acyclic graphs representing the assumed relationships among these variables, SEN exposure, and the outcome of interest will be drawn to identify the variables that will be controlled for to estimate the causal effect of the interventions, using the opensource software, DAGitty ver 3.0.

**Table 5.** Socio-demographic, educational and health characteristics by recorded SEN categories. The table will include means with standard deviations or numbers and row percentages as appropriate, Cleft Lip and/or Palate derived population.

		SEN provision in January Year 1		
Covariate Group	Covariates	None	SEN support	EHCP
**Clinical**	**Cleft severity**			
	Cleft Lip Only			
	Cleft Palate Only			
	Unilateral Cleft Lip and Palate			
	Bilateral Cleft Lip and Palate			
	**Maternal age (years)**			
	**Gestational age (weeks)**			
	**Comorbidities**			
	Mental Behavioural			
	Cancer Blood			
	Chronic infections			
	Respiratory			
	Endocrine Metabolic Digestive Renal Genitourinary			
	Musculoskeletal Skin			
	Neurological Sensory			
	Cardiac conditions			
	**Pre-follow-up: Rate of pre-follow-up outpatient visits (per 1,000 years)**			
	**Pre-follow-up: Rate of pre-follow-up AE visits (per 1,000 years)**			
	**Pre-follow-up: Rate of pre-follow-up Admitted Patient Care visits (per 1,000 years)**			
**Educational**	**Academic cohort (Year 1)**			
	2008/2009			
	2009/2010			
	2010/2011			
	2011/2012			
	2012/2013			
	2013/2014			
	2014/2015			
	2015/2016			
	2016/2017			
	2017/2018			
	2018/2019			
	**Month of birth**			
	Jan			
	Feb			
	Mar			
	Apr			
	May			
	Jun			
	Jul			
	Aug			
	Sep			
	Oct			
	Nov			
	Dec			
	**Early years foundation school profile (z-score)**			
	English			
	Mathematics			
	**Free school meal eligibility**			
	Eligible			
	Not Eligible			
	**School level information: % of SEN**			
	None			
	SEN			
	EHCP			
	**School level information:** **Pupil teacher ratio**			
**Socio-economic**	**Ethnicity** **latest recorded in NPD**			
	Asian Asian British			
	Black Black British Caribbean African			
	Mixed or multiple ethnic groups			
	White			
	Other			
	**English as a first language**			
	Recorded as English			
	Not recorded as English			
	**Income deprivation affecting children index quintile**			
	Most Deprived - 1			
	2			
	3			
	4			
	Least Deprived - 5			
**Outcomes** ** (health)**	**Follow-up: Rate of post-entry days in AE (per 1,000 years)**			
	**Follow-up: Rate of post-entry days in Admitted Patient Care (per 1,000 person-years)**			
	**Follow-up: Rate of post-entry days in APC OR AE (per 1,000 person-years)**			
**Outcomes** ** (education)**	**Medical related absence sessions (per 100,000 sessions)**			
	**Unauthorised absence sessions** **(per 100,000 sessions)**			

Specifically, these covariates, measured at birth or before or at the start of Year One, include: cleft severity (based upon prior literature - ( Fitzsimons *et al.*, 2018) – see Table 1 for ICD10 codes to differentiate cleft severity), comorbidities (categories based upon prior literature ( Hardelid *et al.*, 2014)), gestational age, maternal age, prior hospital contact (unplanned, and outpatient contacts), gender, ethnicity (latest recorded in NPD to reduce missingness), English as a first language, Income Deprivation Affecting Children Index (IDACI) quintile, free school meal eligibility, month of birth, academic cohort (to account for changes in policy over time) and standardised school ready assessments (Early Years Foundation School Profile). Additional school-level variables we aim to include is the proportion of children in the school the child attends in Year One who were recorded as receiving SEN support/EHCP in the previous academic year, and current pupil teacher ratio. 

### Biases

To reduce confounding and other sources of bias affecting observational data, we will adopt a Target Trial Emulation (TTE) framework ( Hernán *et al.,* 2022). TTE enables observational data to be mapped to a hypothetical target experimental trial counterpart by creating the specification of an ideal (pragmatic) trial and using this as a basis to shape the observational study design. TTE consists of one, defining the specifications of a hypothetical target experimental trial of the causal question of interest (including the corresponding effect), two, emulating the specifications of the ideal target trial using observational data and three, estimating the effects of interest using the emulated trial data. The first component of TTE involves defining inclusion/exclusion criteria for entry, a treatment strategy (including time of treatment assignment), follow-up frequency and modality, outcome measures, causal contrasts of interest (estimands) and estimation methods. The second component of TTE involves handling the observational data to emulate the structure of the data that would be gathered in the specified target trial. Finally, the third component of TTE concerns dealing with the inevitable confounding that affects observational data and explicitly outlining the analytical methodology ahead of the data wrangling. In Table 6 we describe the (ideal) target trial one would design to investigate the causal effect of SEN provision on the selected health and educational outcomes in the first year of compulsory education on CLP children and the equivalent emulated trial to be generated from ECHILD.

**Table 6.** Trial emulation to estimate the causal effect of SEN by Year 1 on unplanned hospitalisations by Year 6 in children with cleft lip and/or palate (without other congenital anomalies).

	Ideal target trial	Emulated target trial
**Eligibility criteria**	Geography: England Started Year One between 2008 and 2018. Diagnosed with cleft lip and/or palate prior to Year One Born in England	Geography: England Started Year One in a state school between 2008 and 2018. Identified in HES with cleft lip and/or palate before start of Year One Has a birth record in HES Linked to NPD
**Recruitment** ** period**	Started Year 1 in between the academic years 2008/2009 and 2018/2019	Started Year One between the academic years 2008/2009 and 2018/2019
**Follow-up** ** duration**	From: Randomization to the intervention To: the end of primary school OR loss of follow-up (e.g., emigration) OR death OR end of study	From: January Census in Year One To: the end of primary school OR loss of follow-up in NPD OR death OR end of study/end of data (for HES: 31 August 2019)
**Outcome(s)**	Unplanned hospital utilisation as defined by days in AE or APC Medical related absences as defined using half-day sessions. Unauthorised absences as defined using half day sessions	Unplanned hospital utilisation as defined by days in AE or APC Medical related absences as defined using half-day sessions. Unauthorised absences as defined using half day sessions
**Interventions to** ** be compared**	One of three categories of SEN (none, SEN, EHCP) to be delivered following randomization (between start of reception and end of Year One	One of three categories of SEN (none, SEN, EHCP) as recorded by the January census in Year One
**Causal contrasts**	The average treatment effect of initiating SEN versus non-initiating SEN at all by Year One on the number of unplanned hospital days expressed as a rate ratio. The average treatment effect of initiating EHCP versus initiating SEN by Year One on the number of unplanned hospital days expressed as a rate ratio.	The average treatment effect of recording SEN versus non- initiating SEN at all by Year One on the number of unplanned hospital days expressed as a rate ratio. The average treatment effect of recording EHCP versus recording SEN by Year One on the number of unplanned hospital days expressed as a rate ratio. These estimands will be defined for the whole population and also for the sub-populations of “treated” and “untreated” children, that is the children who were (or were not) recorded to receive the relevant intervention.
**Analysis plan**	Poisson or Negative Binomial Regression (depending on the degree of overdispersion) of the number of events accountings for duration of follow-up. Clustering by school and/or local authority to be dealt with using either mixed effects models or robust inference (e.g., GEE).	Appropriate methods for confounding adjustment (such as regression adjustment and standardisation, or propensity score-based methods) involving Poisson or Negative Binomial Regression (depending on the degree of overdispersion) of the number of events accountings for duration of follow-up. Clustering by school and/or local authority to be dealt with using either mixed effects models or robust inference (e.g., GEE).

The estimands we will target are firstly, the average treatment effect (or average causal effect): this is a causal contrast of average potential outcomes for the whole isolated CLP population, secondly, the average treatment effect in the treated: this is a causal contrast restricted to the “treated”, i.e., those that received SEN and finally, the average treatment effect in the not treated; the causal contrast in those who did not receive SEN in Year One ( Wang *et al.,* 2017).

### Analysis

***Explorative analyses.*** To estimate the representativeness and external validity of the derived cohort, we will compare the following distributions against existing literature, firstly, the rates of CLP children who start Year One between 2008/09 and 2018/19 (CRANE, 2021) and secondly, previously published rates such as school academic attainment ( Fitzsimons *et al.*, 2018). 

To understand whether pupils who are recorded to have received different categories of SEN provision had the chance to be recorded with another category and therefore for the intervention levels to be comparable using the available data (i.e. to assess whether the positivity assumption could be invoked when performing casual inference ( Zhu *et al.*, 2021)), we will examine the distribution of the propensity scores for the recording of SEN support/EHCP across the subgroups of children defined by their observed characteristics. The propensity scores will be predicted using logistic regression, with the covariates mentioned above included as predictors. As there are three categories of SEN (None, SEN Support and EHCP), pairwise propensity score comparisons ( Rassen *et al.*, 2013) will be evaluated for common support between: None vs SEN Support, None vs EHCP and SEN Support vs EHCP. The robustness of the selected propensity score model would be assessed by triangulating the predicted scores with those derived using machine learning methods (such as Classification and Regression Trees) ( Lee Brian *et al.*, 2010).

***Causal inference.*** Once explanatory analyses have been completed, causal inference will be conducted for pairs of interventions which have common support. To account for the distribution of our outcome, that is number of unplanned hospital contacts and number of absence sessions, we will use Poisson (or negative binomial) regression models. To account for differential follow-up time, we will use the logarithm of one of the following as offsets: days between January of Year One and end of follow-up for hospitalisation usage and total number of full sessions between January of Year One and end of follow-up for school absences. The likely clustering of pupils within local authority will be addressed either by fitting mixed effects models or by using robust inference (or both).

We will triangulate complementary estimation methods to address confounding bias due to non-random assignment of SEN provision. We will compare results obtained assuming no-unmeasured confounding (that is, we have data on all the relevant confounders) and assuming instead that we have an instrumental variable (if there is for example variation in SEN provision by local authorities). Our analyses will involve three general approaches.

The first approach will include “traditional” epidemiological methods such as reporting of crude associations (“naïve estimator”) between intervention and outcome, and of conditional associations obtained by fitting appropriate regression models ( Bender, 2009); due to the conditional nature this method, the evaluation of these effects cannot evaluate our causal contracts of interest, such as the average effect on the whole population.

The second approach will involve dealing with measured confounding using outcome-based models ( Smith *et al.*, 2022), such as the parametric g-formula, inverse probability weighting of marginal structural models, and inverse probability weighting regression adjustment. Confidence intervals for these models will be estimated using bootstrapping (1000 replicants). These estimation methods target our estimands of interest, including the average treatment effect, average treatment effect in the treated and the average treatment effect in the not treated (see above).

The third set of methods, instrumental variable analysis, will only be possible if suitable instruments for SEN provision are identified ( Greenland, 2018), for example if there are policy changes in provision that are implemented at different times across local authorities or changes in school policy for example brought about by governance change ( Liu *et al.*, 2020). There is a well-documented change in SEN provision from 2014, which may also allow a difference-in-difference approach to be implemented.

***Missing data.*** Depending upon the proportion of missingness affecting the data and the mechanisms of missingness, we will first use information across data sources to fill the missing information prior to data imputation; for example, using the variable “Sex” held in HES to complement missing values in the NPD variable “Gender”. We will use Imputation using Chained Equations under a missing at random (MAR) assumption to singly imputed missing values, as opposed to multiply imputed, because the imputation will be embedded within the bootstrapping conducted to estimate confidence intervals of point estimates ( Schomaker & Heumann, 2018). All relevant variables (including interactions and non-linearities) will be used to predict missing data including the exposure and the outcome ( Azur *et al.*, 2011).

***Sensitivity analyses.*** To account for uncertainty in the recording of observational data that may lead to measurement errors, we aim to conduct sensitivity analyses. First, we will conduct a sensitivity analysis to mitigate against a delayed recording in SEN provisions, by expanding the exposure window to the first term in Year Two as part of the January census; this analysis will include information collated during Year Six as part of the adjustment set of baseline covariates. Secondly, to understand the driver of unplanned hospitalisation, we will decompose our outcome of unplanned hospitalisation into three categories firstly, the number of days recorded in Accident and Emergency or in Unplanned Admitted Patient Care, secondly, the number of days recorded in Accident and Emergency, and thirdly, the number of days recorded in Unplanned Admitted Patient Care. Similarly, we will examine absences in subgroups defined by whether they were medically related or unauthorised. Finally, we propose to analyse the differences between using recorded child sex (reported by physician in HES at birth) and gender (submitted by parent/carer during school registration in NPD) and produce point estimates tables of the intervention variable when using either measure.

## Ethics and dissemination

Permissions to use linked, de-identified data from Hospital Episode Statistics and the National Public Database were granted by the Department for Education (DR200604.02B) and NHS Digital (DARS-NIC-381972); consent from patients is not required for HES as the data provided by NHS Digital is pseudo-anonymised and reduces identifiability to researchers; further information on opting out of Hospital Episode Statistics for secondary usage can be found here. Ethical approval for the ECHILD project was granted by the National Research Ethics Service (17/LO/1494), NHS Health Research Authority Research Ethics Committee (20/EE/0180) and UCL Great Ormond Street Institute of Child Health’s Joint Research and Development Office (20PE06). Stakeholders (academics, clinicians, educators, and child/young people advocacy groups) will consistently be consulted to refine populations, interventions and outcomes of studies that use the ECHILD dataset to conduct target trial emulation. Scientific, lay and policy briefings will be produced to inform public health policy through partners in the Department of Education and the Department of Health and Social Care.

## Data sharing and access

Aggregate results from the ECHILD dataset will be pre-printed, revised as a protocol, and published. De-identified individual record-level data is currently hosted on the Office for National Statistics Secure Research Service’s data-sharing service. We are grateful to the Office for National Statistics (ONS) for providing the trusted research environment for the ECHILD Database. This does not imply ONS' acceptance of the validity of the methods used to obtain these figures, or of any analysis of the results.

The ECHILD Database uses data from the Department for Education (DfE). The DfE does not accept responsibility for any inferences or conclusions derived by the authors. This work uses data provided by patients and collected by the National Health Service as part of their care and support. Source data can also be accessed by researchers by applying to NHS Digital.

## Conclusions/discussion

his study will contribute towards the understanding of the health and educational impact of Special Educational Needs Provision in a heterogeneous population based upon health needs, specifically those born in England with isolated cleft lip and/or palate. This study will focus on estimating the causal impact of an intervention that can be introduced during a child and young person’s educational journey which may impact their experience of health and education during childhood. 

## Data Availability

No data are associated with this article.
