# Supplementary material for: Early special educational needs provision and its impact on unplanned hospital utilisation and school absences in children with isolated cleft lip and/or palate: a demonstration target trial emulation study protocol using ECHILD
Source: NIHR Open Res. 2023 Oct 24;3:54. [Version 1] doi: 10.3310/nihropenres.13472.1 (PMC11320046; doi:10.3310/nihropenres.13472.1)
Supplement: Peer review history

# Reviewer report: Reviewer response for version 1

**DOI:** 10.3310/nihropenres.14618.r30818

**Published:** 2024-01-17

**Authors:** Chunlin Li

**Affiliations:** 1 School of Biomedical Engineering & Beijing Advanced Innovation Center for Big Data-Based Precision Medicine, Capital Medical University, Beijing, China

This research protocol plan to apply two databases ——National Pupil Database and Hospital Episode Statistics to assess the potential relationship between the special educational needs provision and academic performances of children with cleft lip and palate sand. In general, this protocol written very clearly. However, there may be some areas that need further improvement.

 Firstly, the word “isolated cleft lip and palate” may be a little misleading, I noticed this question has also been mentioned in the second reviewer. At first glance, I thought it was the population with unilateral cleft lip and palate, and I think it will be better with the word “non-syndromic cleft lip and palate”.

 Secondly, speech disorders may trigger a high incidence in patients with cleft palate. However, the incidence rate of this disorder may be slight in patients just with cleft lip. So, I think it will better if research can further investigate the types of special education needs that patients might have receipted. In addition, lower academic performers of patients with cleft lip and palate are caused by various issue, including psychological stress, intelligence, and handedness. These should be considered in the research.

Is the study design appropriate for the research question?

Yes

Is the rationale for, and objectives of, the study clearly described?

Yes

Are sufficient details of the methods provided to allow replication by others?

Yes

Are the datasets clearly presented in a useable and accessible format?

Yes

Reviewer Expertise:

isolated cleft lip and/or palate patients treatment and related brain function investigation

I confirm that I have read this submission and believe that I have an appropriate level of expertise to confirm that it is of an acceptable scientific standard, however I have significant reservations, as outlined above.

# Reviewer report: Reviewer response for version 1

**DOI:** 10.3310/nihropenres.14618.r30814

**Published:** 2023-12-14

**Authors:** Yvonne Wren

**Affiliations:** 1 Bristol Speech and Language Therapy Research Unit, North Bristol NHS Trust, Westbury on Trym, England, UK; 2 Cardiff School of Sport and Health Sciences, Cardiff Metropolitan University, Cardiff, Wales, UK; 3 Cardiff School of Sport and Health Sciences, Cardiff Metropolitan University & Bristol Dental School, University of Bristol, Bristol, UK

The protocol described in this paper makes efficient use of two existing UK national databases- the National Pupil Database and Hospital Episodes Statistics – which provide data on education and health use by children. The plan is to emulate a randomised controlled trial using observational data, which is a sensible approach for research questions, such as this one, which cannot be investigated using trial methodology.

 The population of interest is children born with isolated cleft lip and/or cleft palate, covering all major cleft types but excluding individuals with known syndromes. This is an important population as the population of children born with cleft lip and/or cleft palate in the UK have lower academic outcomes compared to their unaffected peers. Reasons for this have been explored in the literature but our understanding is still limited.

 The paper describes the planned dataset and analyses clearly. However, the justification for the planned analysis is not fully explained. The title states that the purpose is to consider the impact of early special educational needs (SEN) provision on a) unplanned hospital utilisation and b) absences from school, with each of these acting as proxies for health and educational outcomes. The Plain English Summary explains that the intention of this analysis is to see whether children benefit from SEN provision through a reduction in hospital usage and school absences. What is not clear however is why this relationship might exist. As SEN provision is designed to assist children who are at risk of suboptimal educational attainment, it is difficult to understand why this might be associated with unplanned hospital use. Similarly, while school absence could be considered more directly linked to SEN provision in that both are linked to education, the precise link and justification for considering school absences and SEN provision is not provided. 

 In the background section of the abstract, the purpose of SEN provision is given as ‘to improve their speech and language skills’. I think this is misleading. SEN provision can be provided for many reasons. While children born with cleft palate (with or without cleft lip) are at high risk of having problems with their speech – and may indeed receive SEN provision for this – we cannot assume that that is why they are receiving it. An additional analysis could look at the purpose of the SEN provision - of the categorisation of the child’s SEN – in relation to the outcomes, but the analysis as planned must be recognised as being broad SEN provision which could be intended to provide for one or more needs.

 I noticed that the first reviewer queried why the exposure of SEN provision (intervention) was limited to year one and I would also question this. There is a need to have consistency in the dataset of course but it can take a long time for some children to be identified as needing SEN and even longer for provision to be put in place. Moreover, EHCP provision is only for those children with significant need which cannot be provided for within the school and it can take some time for children to be recognised as needing this. It would help to have some additional analysis to inform this decision using data from the National Pupil Database regarding the proportion of children receiving SEN provision and having EHCPs in January of Year one compared to later years to get a sense of how many children might be classified as ‘no SEN’ based on the year one figures but who would be classified as ‘SEN’ in later years.

 It would help readers if the meaning of ‘isolated’ in ‘isolated CLP’ was explained. Generally this is used for those children with CLP who have not been identified with a syndrome. However, as co-occurring conditions are included as confounders, it should be explained how syndromic status is being identified as there is an expectation that some children will have co-occurring conditions in the absence of an identified syndrome. Moreover some children may have CLP as part of a syndrome, but they have not yet been identified as such and that should be acknowledged.

 I was unclear why some of the numbered variables in table 3 were included as presumably the ones of interest are only the first two rows, those relating to planned and unplanned admission?

 Similarly I was unclear in table 4 why row G – number of unauthorised sessions missed during the academic year as pupil is on a holiday….  – is included.

 Aside from the concern about justification of the main purpose of the work, the plans are well-described and replicable and scientifically sound. If a strong rationale for the work can be provided, the analysis and resulting report will be robust.

 Minor points

 On page 4, in the ethics and dissemination paragraph, there should be a space between ‘…ECHILD.’ and ‘Stakeholder…’.

 On page 5, column 2, line 2, ECHILD is written as ECHID. Also the final sentence in this paragraph is unended.

 The covariate ‘free school meal eligibility’ is an important variable but I wondered whether ‘pupil premium status’ would be the relevant term to explore?

 In table 6, year one is sometimes spelt as a word and other times as a number (1). Also there is a closing bracket missing in the second column for the row ‘Interventions to be compared’.

 Page 13, column 2, paragraph 1, there may be a word missing in the fifth line.

 Page 14, column 2, beginning of the Conclusion/discussion section – a letter ‘T’ is missing.

Is the study design appropriate for the research question?

Yes

Is the rationale for, and objectives of, the study clearly described?

No

Are sufficient details of the methods provided to allow replication by others?

Yes

Are the datasets clearly presented in a useable and accessible format?

Yes

Reviewer Expertise:

Cleft lip and/or palate, speech and communication, educational outcomes, psychosocial outcomes, cohort studies, Cleft Collective study

I confirm that I have read this submission and believe that I have an appropriate level of expertise to confirm that it is of an acceptable scientific standard, however I have significant reservations, as outlined above.

# Reviewer report: Reviewer response for version 1

**DOI:** 10.3310/nihropenres.14618.r30721

**Published:** 2023-11-30

**Authors:** Amy L Conrad

**Affiliations:** 1 The Stead Family Department of Pediatrics, The University of Iowa, Iowa City, Iowa, USA

This study utilized two national databases to explore the relationship between provision of special education need (SEN) support and unplanned hospital usage/school absences among students with isolated oral cleft. Careful attention was given to the statistical analyses to emulate an RCT. ( *note* – the statistics are out of my expertise, so my comments will focus on the content and design of the study without commenting on details of the analysis plan).

 The authors provided a very well written and structured study, with ample detail on the dataset, definitions of variables included, and decision points. However, specifics related to the hypothesized casual mechanism between SEN and hospitalizations/absences, anticipated sample size, power to run analyses, and criteria for including additional measures (e.g., academic outcomes) were missing. See below for details.

 Introduction

 - This section is written clearly and concisely, with current literature cited. My only recommendation would be to provide more background information/rational for how it is hypothesized that provision of SEN will impact unplanned hospital utilization/school absences. (As hospitalizations and absences are typically determined by severity of illness, how is it felt that provision of SEN would impact these specific outcomes?)

 Methods

 - Are there any publications or citations related to the two meetings (FLARE and Young Persons Advisory Group) that led to the decision to use school start as the entry point? Interested readers may want additional information on how the meetings were led, how responses were recorded, and how those responses were further narrowed down to the choice of school start as the entry point.

 Intervention

 - While the rationale for limiting group assignment to provision of SEN within the first year of school is provided, this decision may create bias where findings are limited to those with more severe issues (that present early in education) and does not address those with issues that may become more noticeable/impactful over time (e.g., learning disorders). This limitation should be discussed.

 - Are there specific aspects of SEN that are anticipated to have more impact on the outcome measures? For example, could having a teaching assistant have less of an impact than adapted learning programs? Are there plans for a more refined analysis (i.e., more specific coding of elements of SEN [rather than just none vs SEN vs EHCP] that could be tracked?)

 - Gender is listed as a covariate (pg 11), but is not included in Table 5. This variable should be added to the table with the categories that will be used for clarification on if this is “sex determined by doctor at birth” or gender the child reports. (This is discussed a bit on page 14 but should also be included in Table 5.)

 - It is appreciated that the authors discussed why academic performance was not considered as an outcome variable. However, the rationale for omission is not supported with specific estimates of sample size and effect size. For example, prevalence of CLP is estimated at 900/year with 62% expected to have isolated CLP. For those participants that would have academic outcomes (those born between 2003-2008), a sample size of 2,232-2,790 participants with isolated CLP would be expected (i.e., 62% of 900 x 4 to 5 years). Even if the prevalence of SEN was 10%, that would still provide over 200 participants with academic outcomes. The authors do indicate that they would revisit the possibility of including academic outcomes based on numbers with Key State Two data, but no information is given on what baseline sample size would be required to power evaluation of academic outcomes.

 Overall, this study is well structured and outlined. Concerns are minor and are limited to desire for more specifics related to hypothesized casual mechanisms and estimated effect sizes.

Is the study design appropriate for the research question?

Yes

Is the rationale for, and objectives of, the study clearly described?

Partly

Are sufficient details of the methods provided to allow replication by others?

Yes

Are the datasets clearly presented in a useable and accessible format?

Yes

Reviewer Expertise:

Oral Cleft, Academics, Learning Disabilities, Neuropsychological Assessment, Neural Imaging

I confirm that I have read this submission and believe that I have an appropriate level of expertise to confirm that it is of an acceptable scientific standard.
